# Increased Pyrethroid Resistance in Malaria Vectors and Decreased Bed Net Effectiveness, Burkina Faso

**DOI:** 10.3201/eid2010.140619

**Published:** 2014-10

**Authors:** Kobié H. Toé, Christopher M. Jones, Sagnon N’Fale, Hanafy M. Ismail, Roch K. Dabiré, Hilary Ranson

**Affiliations:** Liverpool School of Tropical Medicine, Liverpool, UK (K.H. Toé, C.M. Jones, H.M. Ismail, H. Ranson);; Centre National de Recherche et de la Formation sur Paludisme, Ouagadougou, Burkina Faso (K.H. Toé, S. N’Fale);; Institut de Recherche en Sciences de la Santé/Centre Muraz, Bobo-Dioulasso, Burkina Faso (R.K. Dabiré)

**Keywords:** malaria, parasites, pyrethroid resistance, Anopheles gambiae, mosquitoes, vector control, bed nets, bed net effectiveness, Burkina Faso

## Abstract

Malaria control is dependent on insecticides. Increases in prevalence of insecticide resistance in malaria vectors across Africa are well-documented. However, few attempts have been made to quantify the strength of this resistance and link it to the effectiveness of control tools. Using quantitative bioassays, we show that in Burkina Faso pyrethroid resistance in *Anopheles gambiae* mosquitoes has increased in intensity in recent years and now exceeds 1,000-fold. In laboratory assays, this level of resistance renders insecticides used to impregnate bed nets ineffective. Thus, the level of personal and community protection afforded by long-lasting insecticide-treated net campaigns will probably be reduced. Standardized methods are needed to quantify resistance levels in malaria vectors and link these levels to failure of vector control methods.

## Abstract 2

This new resistance will have serious effects on malaria control.

Long-lasting insecticide–treated bed nets (LLINs) have been shown repeatedly to provide protection against malaria transmission in Africa and reduce childhood mortality rates by ≈20% (*1*). Distribution of LLINs has increased over the past decade, and an estimated 54% of households at risk for malaria in sub-Saharan Africa have ≥1 LLIN. This factor has been a major contributor in reducing malaria incidence; the estimated malaria mortality rate for Africa has decreased by ≈49% since 2000 (*2*). These advances are now threatened by rapid selection and spread of resistance to insecticides in malaria vectors (*3*). Resistance to pyrethroids, the only class of insecticides available for use on LLINs, is now widespread in *Anopheles gambiae* and *An. funestus* mosquitoes, the major malaria vectors (*4*).

To standardize monitoring for insecticide resistance, the World Health Organization (WHO) has developed simple bioassays that use filter papers impregnated with insecticide at a predefined diagnostic dose. A population is described as resistant to an insecticide if a mortality rate >90% is observed in these tests (*5*). These assays are useful for detecting resistance when it first appears in the population. However, these assays do not provide any information on the strength of this resistance. This information is crucial for assessing the likely effect of this resistance on effectiveness of vector control tools. The Global Plan for Insecticide Resistance Management in Malaria Vectors (*3*) recommends that all malaria-endemic countries monitor insecticide resistance in local vectors. However, because the correlation between results of diagnostic dose assays and control effectiveness remains undefined, simple detection of resistance in a mosquito population is not sufficient evidence to implement a change in insecticide policy.

In this study, we used variants of WHO assays and bottle assays of the Centers for Disease Control and Prevention (CDC) (Atlanta, GA, USA) to quantify the level of pyrethroid resistance in a population of *An. gambiae* mosquitoes from Burkina Faso over a 3-year period. A high level of resistance was observed. The lack of comparator data from across Africa makes it impossible to conclude whether the pyrethroid resistance levels seen in Burkina Faso are atypical. However, these data should raise concerns for malaria control across Africa because we demonstrate that this level of resistance is causing operational failure of the insecticides used in LLINs.

## Materials and Methods

The study site was in Vallée de Kou (Bama) in southwestern Burkina Faso, ≈25 km from the city of Bobo-Dioulasso. It consists of 7 small villages (area 1,200 hectares) and has been a major rice cultivation site since the 1970s. The area is surrounded by cotton-, rice-, and vegetable-growing areas in which insecticide use is intensive (*6*). Multiple rounds of collections of third and fourth instar *Anopheles* spp. larvae were performed in a 1-km^2^ radius from village 7 during June–July 2011, October 2011, June 2012, and July–October 2013. Mosquitoes from each collection round were pooled and reared to adults in insectaries at the Institut de Recherche en Sciences de la Sante/Centre Muraz in Bobo-Dioulasso or the Centre National de Recherche et de Formation sur le Paludisme (CNRFP) in Ouagadougou. Species were identified for a subset of mosquitoes from each collection round by using the Sine 200 PCR (*7*).

Non–blood fed *An. gambiae* female mosquitoes (3–5 days old) were tested with 5 insecticides in 4 insecticide classes: 0.75% permethrin (type I pyrethroid) and 0.05% deltamethrin (type II pyrethroid); 4% DDT (organochlorine); 0.1% bendiocarb (carbamate); and 1% fenitrothion (organophosphate) by using WHO susceptibility tests (*8*). Each batch of insecticide-impregnated papers was tested against mosquitoes of the *An*. *gambiae* Kisumu laboratory strain (insecticide-susceptible) at the CNRFP bioassay laboratory for quality control. Approximately 100 mosquitoes (4 replicates of 25 mosquitoes) were used per test (*5*). The average mortality rate and binomial confidence interval were calculated per insecticide (*9*).

In 2011 and 2012, the 50% lethality time (LT_50_) for the VK7 strain of *An. gambiae* mosquitoes was determined by varying the length of exposure time (60–600 min). The mean mortality rate was recorded per time point, and the LT_50_ was estimated by fitting a logistic regression model by using logit-transformed probabilities (*10*) in R statistical software (http://www.r-project.org).

In 2013, CDC bottle bioassays were used to quantify the level of resistance to deltamethrin. Glass 250-mL bottles were coated with different concentration of deltamethrin ranging from 3.125 μg/mL to 125 μg/mL at CNRFP. Bottles were prepared according to CDC guidelines (*11*). Female mosquitoes (3–5 days) were aspirated into bottles for 1 h and subsequently transferred to insecticide-free paper cups for 24 h of observation. Four to six replicates were performed for each concentration and for the control bottles (impregnated with acetone). Equivalent age mosquitoes of the Kisumu strain were exposed to various insecticide concentrations (range 0.001 μg/mL–0.5 μg/mL). The 50% lethal dose (LD_50_) was determined by using R statistical software.

A subset of LLINs that were distributed during the 2010 national distribution campaign were collected directly from houses in 2012; householders were given a new LLIN as a replacement. Only nets reportedly washed ≤5 times were included in the study. New net samples of the same type were also obtained from the population or from local markets. Six types of nets were tested: PermaNet 2.0 (deltamethrin coated on polyester; Vestergaard, Lausanne, Switzerland); Interceptor (α-cypermethrin coated on polyester; BASF, Florham Park, NJ, USA); DawaPlus (deltamethrin coated on polyester; TANA Netting Ltd., Bangkok, Thailand); NetProtect (deltamethrin incorporated into polyethylene; BESTNET, Kolding, Denmark); PermaNet 3.0 (deltamethrin coated on polyester with strengthened border side panels and deltamethrin and piperonyl butoxide incorporated into a polyethylene roof; Vestergaard); and Olyset (permethrin incorporated into polyethylene; Sumitomo Chemical Co., Ltd., Osaka, Japan)..

Cone bioassays were performed according to WHO procedures (*12*) by using non–blood fed VK7 mosquitoes (3–5 days old) (obtained from larvae collection during October–December 2012) and Kisumu strain mosquitoes. Approximately 60 mosquitoes were assessed per net by using net samples from 2 sides and the top (20 mosquitoes/net sample). Mosquitoes were exposed to the insecticide for 3 min. Knockdown was recorded after 60 min, and the mortality rate was determined 24 h later. Mortality rates after exposure to each net were compared for wild-type and laboratory susceptible (laboratory raised) mosquitoes by using the Fisher exact test.

High-performance liquid chromatography was used to measure the insecticide content of 12 nets. Triplicate samples were tested from each net, and insecticide was extracted from five 8-cm^2^ disks for each sample by vortexing them in acetone. A 10-μL aliquot was injected onto a reverse-phase, 250 mm, C18 column (Acclaim 120; Dionex, Sunnyvale, CA, USA). Separation was achieved by using a mobile phase of methanol/water (90:10 vol/vol) and at flow rate of 1 mL/min. Pyrethroid elution was monitored by absorption at 232 nm and quantified by peak integration (Chromeleon; Dionex). The quantity of pyrethroid insecticide was determined from a standard curve established with known concentration of pyrethroid insecticide.

## Results

**Figure 1:**
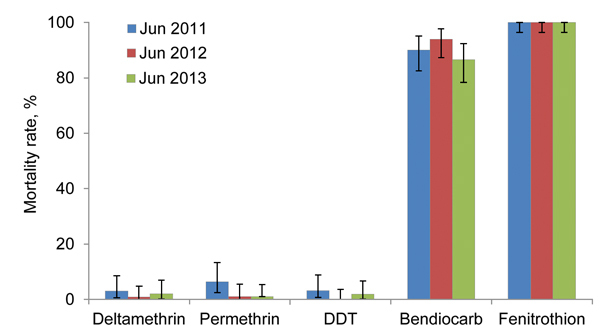
Results of World Health Organization (WHO) susceptibility tests for *Anopheles gambiae* VK7 mosquitoes, Burkina Faso. Adult female mosquitos were exposed to the WHO diagnostic dose of insecticides for 1 h, and mortality rates were recorded 24 h later. Error bars indicate 95% binomial CIs for 3 consecutive years (2011–2013) of sampling.

All *An. gambiae* VK7 mosquitoes collected were the M form, except for those collected during October 2011 and June–July 2013, of which the M form comprised 92% (315/335) and 90% (258/287) of the *An. gambiae* sensu lato populations, respectively. Susceptibility to 5 insecticides was assessed in adults emerging from VK7 strain larval collections in 3 successive years. *An. gambiae* mosquitoes remained fully susceptible to fenitrothion and showed a high mortality rate to bendiocarb (86.5% in June 2013) but low mortality rates to DDT (range 0%–3%) and for the pyrethroids deltamethrin and permethrin (range 1%–6%) However, no significant differences were found between results of the 3 successive years (p = 0.055) (Figure 1).

**Figure 2:**
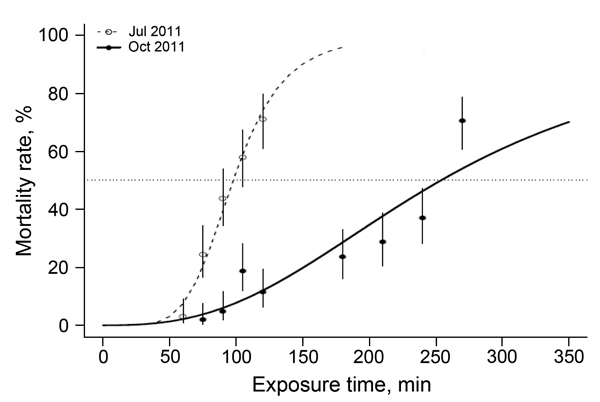
Time-response curves for *Anopheles gambiae* VK7 mosquitoes, Burkina Faso, July–October 2011. Adult females were exposed to 0.05% deltamethrin according to World Health Organization standard protocols. Time-response curves were fitted to data by using a regression logistic model and R software (http://www.r-project.org/). Dotted line indicates 50% mortality rate. Error bars indicate 95% binomial CIs for each time point. The 50% lethality times were 1 h 38 min for July and 4 h 14 min for October, which indicates an October:July resistance ratio increase of 2.6-fold.

Initially, the strength of resistance was assessed by determining the LT_50_ for deltamethrin. In July 2011, an LT_50_ of 1 h 38 min (95% CI 1 h 34 min–1 h 42 min) was obtained but this value increased to 4 h 14 min (95% CI  3 h 53 min–4 h 36 min) in October of the same year (Figure 2), which is a 2.6-fold increase in only 4 months. An accurate LT_50_ could not be determined for samples collected in June 2012. The longest exposure time of 600 min (10 h) showed a mortality rate of 26% (95% CI 17.85%–35.50%), which extrapolates to an LT_50_ of 21 h 55 min (95% CI 14 h 3 min–34 h 14 min). The estimated LT_50_ for the Kisumu strain was <2 min (*13*). This time equates to resistance ratios in the field population versus the susceptible (laboratory raised) strain of 54- fold, 141-fold, and 730-fold in the 3 successive sampling periods.

**Figure 3:**
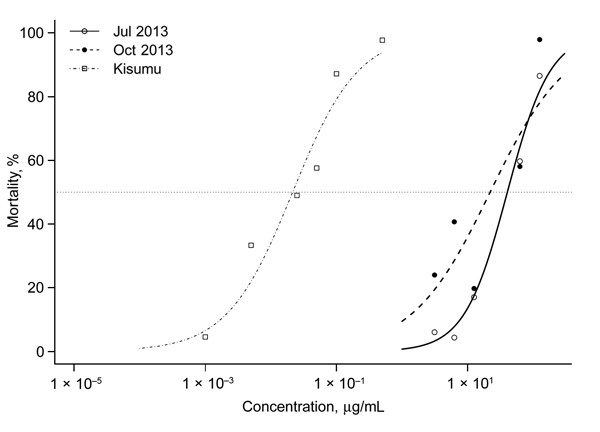
Dose-response curves for 3- to 5-day-old *Anopheles gambiae* VK7 female mosquitoes and Kisumu laboratory strain mosquitoes (insecticide-susceptible), Burkina Faso. Mosquitoes were exposed to different concentrations of deltamethrin in 250-mL glass bottles for 1 h. Dose-response curves were fitted to data by using a regression logistic model and R software (http://www.r-project.org/). Dotted line indicates 50% mortality rate. 50% lethality concentrations were 38.787 μg/mL (95% CI 32.993 μg/mL–46.062 μg/mL) in July 2013, 21.547 μg/mL (95% CI 15.771 μg/mL–31.223 μg/mL) in October 2013, and 0.021 μg/mL (95% CI 0.015 μ /mL–0.029 μg/mL) for the Kisumu strain.

Because the resistance level exceeded the threshold at which accurate LT_50_ levels were obtainable, in 2013, a variation of the CDC bottle bioassays was used to calculate the strength of resistance. Mosquitoes collected in July 2013 had an LD_50_ of 38.79 μg/mL (95% CI 32.99 μg/mL−46.06 μg/mL). The LD_50_ estimate for October was lower (21.55 μg/mL, 95% CI 15.77 μg/mL–31.22 µg/mL) and showed greater variation (Figure 3). By comparison, the LD_50_ for the insecticide-susceptible Kisumu strain calculated by using the same method was 0.021 μg/mL (95% CI 0.015 µg/mL–0.029 µg/mL). This value is equivalent to VK7:Kisumu resistance ratios of 1,847:1 for July and 1,026:1 for October.

**Figure 4:**
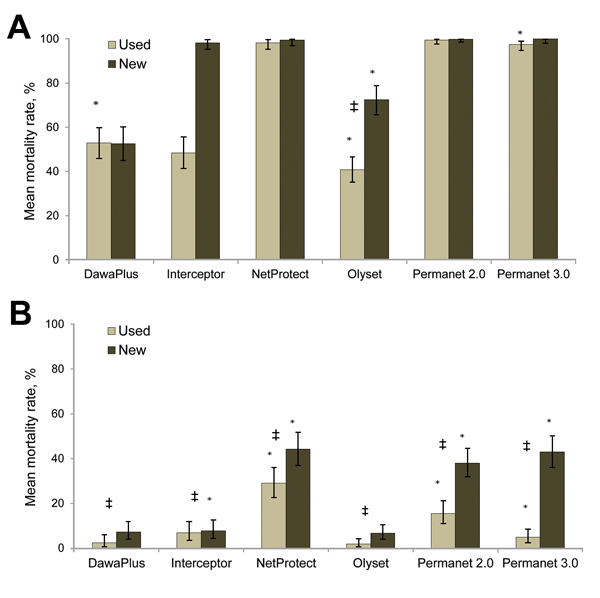
Mean mortality rates 24 h after exposure to new and used long-lasting insecticide–treated bed nets for A) *Anopheles gambiae* Kisumu laboratory strain mosquitoes (insecticide-susceptible) and B) *An. gambiae* VK7 mosquitoes, Burkino Faso. Error bars indicate 95% binomial CIs for the average of net type. *Indicates significant variation between independent nets of each type (p<0.05), ‡Indicates a significant difference between new and currently long-lasting insecticide-treated bed nets (p<0.05).

The efficacies of 6 types of LLINs distributed as part of the National Malaria Control Program of Burkina Faso were assessed against the Kisumu and VK7 mosquito strains to assess the effect of resistance on LLIN effectiveness in a standardized WHO bioassay. New nets and nets that had been in use in the field for ≈2 years were assessed. Only 4 of 6 new nets showed 100% mortality rates against the Kisumu strain (Figure 4, panel A); a fifth net (Olyset) satisfied only the knockdown criteria. When used nets were tested, only 3 nets (Permanet 2.0, Permanet 3.0, and NetProtect) satisfied the WHO criteria (mortality rate >80% and knockdown rate >95%). When we evaluated the nets against VK7 mosquitoes, none of the nets satisfied the knockdown criteria and mean mortality rates were <50% for all new nets and used LLINs tested; mortality rates were lower for used nets than for all types of new nets (Figure 4, panel B).

Because PermaNet 3.0 nets have the synergist piperonyl butoxide incorporated into the net roof, we compared bioassay data for the roof and sides. Mortality rates were significantly higher when mosquitoes were exposed to the roof of the net when new nets were tested (p = 0.011) but not when used nets were tested (p = 0.20).

Low mortality rates observed in cone bioassays, even against susceptible mosquitoes, led us to investigate the amount of insecticide that could be extracted from LLINs used in the bioassays. Analysis by high-performance liquid chromatography was performed for net types that did not satisfy WHO criteria for susceptible mosquitoes; Permanet 2.0 was used as a control. We included data only for nets in which insecticide was coated onto the surface (rather than incorporated into the fibers) because the acetone extraction method used is not efficient in extracting insecticide from the fibers. In each case, higher levels of insecticide were extracted from new nets than from used nets (p<0.05, by 2-tailed *t* test) except for DawaPlus, for which the amount of active ingredient was ≈0 for used and new nets (Table 1). Less than 12% of the target concentration of insecticide could be extracted from LLINs that induced a mortality rate <80% in the Kisumu strain. In general, the expected concentration of insecticide was isolated from LLINs, which showed the target mortality rate of 80% for cone bioassays.

**Table 1:** Characteristics of insecticide extracted from long-lasting insecticide–treated bed nets, Burkina Faso*

Net type	Condition	No. tested	Active ingredient concentration, mg/m^2^ (SD)	Target concentration, mg/m^2^	Mortality rate, %, for *Anopheles gambiae* Kisumu laboratory strain mosquitoes (95% CI)†
DawaPlus	Used	3	0	80	52.43 (45.37–59.41)
DawaPlus	New	2	1.31 (1.60)	80	51.30 (41.80–60.73)
Interceptor	Used	3	22.2 (18.56)	200	48.48 (41.30–55.67)
Interceptor	New	2	223.95 (43.58)	200	97.65 (93.30–99.51)
PermaNet 2.0	Used	2	66.3 (1.82)	55	100.00 (97.02–100.00)

*Active ingredient concentration was determined by high-performance liquid chromatography. Mortality rate was determined after a 3-min exposure. †Insecticide susceptible.

## Discussion

Monitoring *Anopheles* spp. vectors for susceptibility to insecticides is recommended by WHO in all countries that use LLINs or indoor residual spraying for malaria control (*3*). Most countries in Africa that have implemented resistance monitoring since 2010 have detected pyrethroid resistance in some regions. Many countries have detected resistance to multiple insecticide classes (www.irmapper.com). Ideally, the first detection of resistance should elicit a change in insecticide class as part of a proactive resistance management program. However, because only pyrethroids are currently available for impregnating bed nets, and use of alternative insecticide classes for indoor residual spraying often result in higher program costs (*14*,*15*), resistance management options are severely limited. Thus, data obtained from routine resistance monitoring should be sufficient to make evidence-based decisions on insecticide-based vector control strategies. Use of only diagnostic dose assays alone can mask major changes in the strength of resistance. This finding can be seen in the current study, in which no major difference was seen in pyrethroid mortality rates over a 3-year period with a fixed exposure of insecticide. However, when exposure time or concentration was varied, increases in the strength of the resistance were observed.

We used 3 bioassays to quantify the strength of the resistance and link this strength to the effectiveness of current vector control tools. Each of these methods has limitations, which are summarized in Table 2. In this study, we did not use a tunnel test, in which mosquitoes are exposed overnight to a holed net, with a guinea pig as bait, and the mortality rate and blood-feeding inhibition are measured (*12*). It would be useful to determine if the longer exposure time used in the tunnel test resulted in higher mortality rates, although given the long exposure time required to achieve the LT_50_ with impregnated papers, it is expected that the LLINs would also fail a tunnel test against the VK7 mosquito population.

**Table 2:** Alternative field bioassay methods for assessing strength of insecticide resistance*

Method	Brief description	Advantages	Disadvantages
Time-response curves (LT_50_)	Exposure to fixed concentration of insecticide for varying periods	Can be performed by using diagnostic dose filter papers available from WHO; simple to perform	Not appropriate for highly resistant mosquito populations in which long exposure times required
Dose-response curves (LD_50_)	Exposure to varying concentrations of insecticide for a fixed period	Can be readily adapted for populations of different resistance status by varying concentration of insecticide used	Challenging to accurately measure small quantities of insecticide needed for some pyrethroid insecticides; if bottles are reused, stringent washing conditions are needed
Cone bioassays on treated surfaces	Exposure to field dose of insecticide for fixed period	Concentration of insecticide being evaluated is the field dose	Mosquitoes can avoid exposure by resting on side of cones, particularly for new preparations of some pyrethroids.

*For all assays, use of age-standardized mosquitoes and inclusion of a 24-h recovery period to capture metabolic resistance mechanisms (which can be slower to act) is recommended. LT_50_, 50% lethality time; WHO, World Health Organization; LD_50_, 50% lethal dose.

There is a need for agreement on a consensus method for resistance monitoring, together with clear guidelines for interpreting the operational value of the results and recommended courses of action. This method would not necessarily replace diagnostic dose assays, which are valuable for detecting the prevalence of resistance in a population, but would instead provide a quantitative estimate of the strength of resistance that is linked to predicted control failure. Such assays are common practice in the agricultural sector and a cross-sectorial approach would be invaluable for improving resistance monitoring in malaria control.

The resistance levels we report in the current study are alarming. Because few studies have attempted to quantify resistance strength in field populations, it is difficult to know if this extreme resistance phenotype is exceptional or symptomatic of the status of pyrethroid resistance in malaria vectors in Africa. Two other studies have used the LT_50_ method to assess the strength of resistance to pyrethroids in field populations compared with susceptible (laboratory raised) strains. In 2011, deltamethrin resistance ratios of 138-fold were recorded in Tiassalé, Côte d’Ivoire (*13*) and 292-fold in Jinja, Uganda (*16*). Thus, to our knowledge, deltamethrin resistance levels of 730-fold in 2012 (estimated by LT_50_) and >1,000 fold in 2013 (estimated by LD_50_) reported in the current study are the highest in the published literature.

This level of resistance will almost certainly affect the effectiveness of vector control. We demonstrate that the insecticide resistance of VK7 mosquitoes severely affected the performance of LLINs in standardized laboratory bioassays. In Kenya, pyrethroid-resistant mosquitoes were found resting inside holed LLINs and, when tested by cone bioassays, these LLINs were also found to be ineffective at killing local vectors (*17*). Linking resistance strength with increases in malaria transmission is currently not possible but is a key priority for further studies. No data on the strength of pyrethroid resistance in *An. funestus* mosquitoes in southern Africa in 2000 are available. This resistance has been widely accredited with causing control failure that resulted in a dramatic increase in malaria cases (*18*).

Finally, it is vital to recognize that insecticide resistance is not the only cause of reduced effectiveness of vector control tools. In the current study, we showed that cone bioassays for new and used LLINS were less effective at killing the field-caught *An. gambiae* mosquitoes than they were against a standard susceptible (laboratory raised) strain, which provided additional evidence for the effect of resistance. However, we also found that 2 brands of the LLINs (Olyset and DawaPlus) showed poor performance against the susceptible mosquito strain, and another LLIN (Interceptor) showed adequate performance only when new nets were used. Although these data were obtained for a small sample set, they are a cause for concern and must be investigated further.

## References

[1] Lim SS, Fullman N, Stokes A, Ravishankar N, Masiye F, Murray CJ, Net benefits: a multicountry analysis of observational data examining associations between insecticide-treated mosquito nets and health outcomes. PLoS Med. 2011;8:e1001091 . 10.1371/journal.pmed.1001091 21909249 PMC3167799

[2] World Health Organization. World malaria report: 2013. Geneva: The Organization; 2013.

[3] World Health Organization. Global plan for insecticide resistance management in malaria vectors (GPIRM). Geneva: The Organization; 2012.

[4] Ranson H, N’Guessan R, Lines J, Moiroux N, Nkuni Z, Corbel V. Pyrethroid resistance in African anopheline mosquitoes: what are the implications for malaria control? Trends Parasitol. 2011;27:91–8 . 10.1016/j.pt.2010.08.004 20843745

[5] World Health Organization. Test procedures for monitoring insecticide resistance in malaria vector mosquitoes. Geneva: The Organization; 2013.

[6] Dabiré KR, Diabate A, Djogbenou L, Ouari A, N'Guessan R, Ouedraogo JB, Dynamics of multiple insecticide resistance in the malaria vector *Anopheles gambiae* in a rice growing area in south-western Burkina Faso. Malar J. 2008;7:188 . 10.1186/1475-2875-7-188 18817564 PMC2564969

[7] Santolamazza F, Mancini E, Simard F, Qi Y, Tu Z, della Torre A. Insertion polymorphisms of SINE200 retrotransposons within speciation islands of *Anopheles gambiae* molecular forms. Malar J. 2008;7:163 . 10.1186/1475-2875-7-163 18724871 PMC2546427

[8] World Health Organization. Report of the WHO informal consultation on test procedures for insecticide resistance monitoring in malaria vectors, bio-efficacy and persistence of insecticides on treated surfaces. Geneva: the Organization; 1998.

[9] Newcombe RG. Two-sided confidence intervals for the single proportion: comparison of seven methods. Stat Med. 1998;17:857–72 . 10.1002/(SICI)1097-0258(19980430)17:8<857::AID-SIM777>3.0.CO;2-E 9595616

[10] Müller P, Chouaibou M, Pignatelli P, Etang J, Walker ED, Donnelly MJ, Pyrethroid tolerance is associated with elevated expression of antioxidants and agricultural practice in *Anopheles arabiensis* sampled from an area of cotton fields in northern Cameroon. Mol Ecol. 2008;17:1145–55 . 10.1111/j.1365-294X.2007.03617.x 18179425

[11] Centers for Disease Control and Prevention. Guidelines for evaluating insecticide resistance in vectors using the CDC bottle bioassay, 2013 [cit5ed 2014 Jun 18]. http://www.cdc.gov/malaria/resources/pdf/fsp/ir_manual/ir_cdc_bioassay_en.pdf.

[12] World Health Organization. Guidelines for laboratory and field testing of long-lasting insecticidal treated nets. Geneva: The Organization; 2005.

[13] Edi CV, Koudou BG, Jones CM, Weetman D, Ranson H. Multiple-insecticide resistance in *Anopheles gambiae* mosquitoes, southern Côte d’Ivoire. Emerg Infect Dis. 2012;18:1508–11 . 10.3201/eid1809.120262 22932478 PMC3437712

[14] Laxminarayan R, Chow J, Shahid-Salles S. Intervention cost-effectiveness: overview of main messages. In: Jamison DT, Breman JG, Measham AR, Alleyne G, Claeson M, Evans DB, et al., editors. Disease control priorities in developing countries. 2nd ed. Washington (DC): The World Bank; 2006. p. 35–86. 21250358

[15] Conteh L, Sharp BL, Streat E, Barreto A, Konar S. The cost and cost-effectiveness of malaria vector control by residual insecticide house-spraying in southern Mozambique: a rural and urban analysis. Trop Med Int Health. 2004;9:125–32 . 10.1046/j.1365-3156.2003.01150.x 14728616

[16] Mawejje HD, Wilding CS, Rippon EJ, Hughes A, Weetman D, Donnelly MJ. Insecticide resistance monitoring of field-collected *Anopheles gambiae* s.l. populations from Jinja, eastern Uganda, identifies high levels of pyrethroid resistance. Med Vet Entomol. 2013;27:276–83 . 10.1111/j.1365-2915.2012.01055.x 23046446 PMC3543752

[17] Ochomo EO, Bayoh NM, Walker ED, Abongo BO, Ombok MO, Ouma C, The efficacy of long-lasting nets with declining physical integrity may be compromised in areas with high levels of pyrethroid resistance. Malar J. 2013;12:368 . 10.1186/1475-2875-12-368 24156715 PMC4016513

[18] Hargreaves K, Koekemoer LL, Brooke BD, Hunt RH, Mthembu J, Coetzee M. *Anopheles funestus* resistant to pyrethroid insecticides in South Africa. Med Vet Entomol. 2000;14:181–9 . 10.1046/j.1365-2915.2000.00234.x 10872862

